# Phytochemistry and Antihyperglycemic Potential of *Cistus salviifolius* L., Cistaceae

**DOI:** 10.3390/molecules27228003

**Published:** 2022-11-18

**Authors:** Maja Hitl, Katarina Bijelić, Nebojša Stilinović, Biljana Božin, Branislava Srđenović-Čonić, Ljilja Torović, Nebojša Kladar

**Affiliations:** 1 Department of Pharmacy, Faculty of Medicine, University of Novi Sad, 21000 Novi Sad, Serbia; 2 Department of Pharmacology and Toxicology, Faculty of Medicine, University of Novi Sad, 21000 Novi Sad, Serbia; 3 Center for Medical and Pharmaceutical Investigations and Quality Control (CEMPhIC), Faculty of Medicine, University of Novi Sad, 21000 Novi Sad, Serbia; 4 Institute of Public Health of Vojvodina, 21000 Novi Sad, Serbia

**Keywords:** extracts, essential oil, HPLC-DAD, GC-MS, antioxidant, antihyperglycemic

## Abstract

*Cistus salviifolius* has been previously reported as a traditional remedy for hyperglycemia. However, the plant has been scarcely investigated from scientific point of view. Thus, the aim was to examine the chemical composition and to evaluate its antioxidant and antihyperglycemic potential in vitro. Aqueous and ethanolic extracts were evaluated for total phenolic, tannin, and flavonoid content using spectrophotometric methods. Detailed chemical characterization was performed by high-performance liquid chromatography (HPLC-DAD). The volatile organic compounds (VOCs) profile was assessed by gas chromatography technique. The potential in diabetes treatment was evaluated through tests of free radicals neutralization, inhibition of lipid peroxidation process, and test of ferric ion reduction; activity in tests of inhibition of α-amylase, α-glucosidase and dipeptidyl peptidase-4 was also evaluated. High content of phenolics (majority being tannins) was detected; detailed HPLC analysis revealed high content of gallic acid, followed by rutin, chlorogenic and caffeic acids. The VOCs analysis determined sesquiterpene hydrocarbons and oxygenated sesquiterpenes as the main groups of compounds. The assays classified extracts as potent neutralizers of 2,2-diphenyl-1-picrylhydrazil and nitroso radicals formation and potent inhibitors of α-amylase and α-glucosidase. In conclusion, *Cistus salviifolius* represents a rich source of phenolics and essential oil with sesquiterpenes. The established results suggested its promising antioxidant and antihyperglycemic activities.

## 1. Introduction

Genus *Cistus* comprises of species classified into three subgenera: *Cistus*, *Leucocistus*, and *Halimioides*. These plants are usually perennial shrubs, with small, simple, and harsh leaves and actinomorphic flowers, ranging from white to pink or purple color depending on the subgenus [1]. Representatives of the genus inhabit the Mediterranean region, growing in hostile conditions (rocky, dry, or infertile terrain) and are sometimes called rock-roses [2]. They are even found at former mining sites, as pioneers of inhabitation, where they can serve for soil remediation from toxic elements [3,4]. Additionally, the ability to inhabit terrains after forest fires is a consequence of the seed’s hard coat and stimulation of germination by high temperatures [5,6]. Due to that, they are sometimes considered pyrrophytae (Greek: phyrro—fire, flames; phyton—plant), not to be mistaken with the algal species [6].

The geographical and ecological occurrence of genus *Cistus* representatives suggests an abundancy in specific secondary metabolites which stimulate growth in such harsh conditions [7]. However, these compounds have also found application as nutraceuticals, pharmaceuticals, and ingredients in perfumes, as well as in the chemical industry [7,8]. Various species of the *Cistus* genus are also present in the traditional medicine of Mediterranean countries. Numerous conditions and diseases are reported to be treated with these herbs, with the respiratory and digestive issues being the most common [2]. Some preparations have been recognized as of commercial significance, labdanum (a resin from *C. ladanifer* species) possibly being the most famous for its application in the perfume industry [9].

*Cistus salviifolius* is a representative of the subgenus *Leucocistus*, characterized by white flowers. Additionally, species of this subgenus are reported to contain high amounts of ellagitannins, while flavonoids are only scarcely present. The name “salviifolius” is given based on a specific odor of fresh leaves which resembles sage; besides this, some morphological similarity of leaves can also be noted [1]. This *Cistus* species is relatively poorly investigated for its medicinal and pharmaceutical potential. On the other hand, its traditional application has been recorded, mainly in some digestive disorders, both in prevention and treatment. Among them, the treatment of diarrhea and the prevention and treatment of hyperglycemia are mentioned [2,10]. According to these facts, the aim of the present study was to determine the chemical compounds present in *Cistus salviifolius* extracts and to evaluate their antioxidant and antihyperglycemic potential. Due to several previously published papers reporting results derived of a limited number of tests, this investigation opted for testing several different, yet complementary systems which provide better insight into the antioxidant and antihyperglycemic properties of *Cistus*.

## 2. Results

### 2.1. Preliminary Chemical Characterization

The results of the preliminary chemical analysis are presented in Table 1. Herbal samples can be characterized as high in phenolic content, where the majority of phenolic compounds were tannins.

### 2.2. High-Performance Liquid Chromatography Coupled to Diode Array Detector (HPLC-DAD) Chemical Profiling

**Figure 1:**
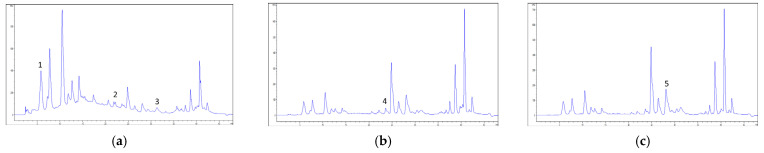
High-performance liquid chromatography coupled to diode array detector (HPLC-DAD) chromatograms of sample P-Et (herb collected at Petaloudes location, extraction with ethanol) with detection at: (**a**) 280 nm, (**b**) 330 nm and (**c**) 350 nm. Detected compounds corresponding to peaks: 1—gallic acid, 2—caffeic acid, 3—trans-cinnamic acid, 4—chlorogenic acid, 5—rutin.

The results of the detailed chemical analysis (Figure 1) are presented in Table 2.

A high content of gallic acid was recorded in both samples. Rutin was the second most abundant compound, followed by chlorogenic and caffeic acids. The presence of *trans*-cinnamic acid was confirmed only in ethanolic extracts. However, several compounds were not detected in tested extracts.

### 2.3. Chemical Analysis of Volatile Organic Compounds (VOCs)

**Figure 2:**
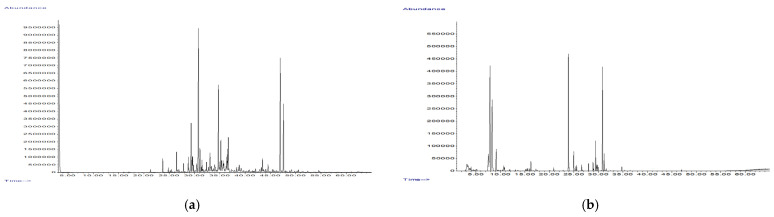
GC-MS chromatograms of essential oil isolated from sample P (**a**) and volatiles isolated by HSS technique from sample P (**b**).

The results of VOCs analysis in both dried leaves and essential oils (EOs) of *Cistus salviifolius* (Figure 2) are given in Table 3. Concerning the EOs, the yield of extraction was 0.09% for the E and 0.09% for the P sample.

The pleasant aroma of *C. salviifolius* leaves in their fresh state suggests the presence of VOCs as secondary biomolecules in the plant. The present study opted for the comparison of results of the VOCs content as some differences between dried raw herbal material and isolated EO can occur as a result of increased temperature used for EO hydrodistillation. In both collected plant samples, regardless of the method of sample preparation, sesquiterpene hydrocarbons were the most abundant group of compounds (ranging 65.3–79% of total VOC content), followed by oxygenated sesquiterpenes (9.95–11.53% of total VOC content). However, notable differences could be observed between the chemical profile of dried leaves and EO, where, in the former, notable amounts of all subclasses of monoterpenes, as well as aliphatic compounds were recorded. The EOs from both samples contained notably higher amount of sesquiterpene hydrocarbons, as well as aromatic sesquiterpene hydrocarbons. Furthermore, aliphatic compounds were not recorded in the investigated EOs.

The single most-abundant component (approximately 40%, ranging 39.78–41.09%) in all tested samples was germacrene D, followed by another sesquiterpene hydrocarbon, (E)-β-farnesene (range: 16.39–19.67%) and an oxygenated sesquiterpene, caryophyllene oxide (range: 5.66–9.77%), with the latter two in somewhat higher percent in dried leaves than in EO.

### 2.4. Antioxidant Potential

The testing of antioxidant potential was performed using five different assays. The results are presented in Table 4. The antioxidant activity was prominent in tests scavenging synthetic- 2,2-diphenyl-1-picrylhydrazil (DPPH) and naturally occurring nitroso (NO) radicals, while weaker activity was recorded in other assays.

### 2.5. Antihyperglycemic Potential

The extracts of *Cistus salviifolius* exhibited potent activity against enzymes α-amylase and α-glucosidase, while weak activity against DPP-4 enzyme was observed. The results are presented in Table 5.

In the present study, ethanolic extracts were more potent than the aqueous ones in α-amylase and α-glucosidase inhibition. When compared to acarbose, ethanolic extracts were approximately equally active against α-amylase; however, all samples were more potent than acarbose in α-glucosidase inhibition. To the best of our knowledge, this is one of the first papers testing the effect of *Cistus* extracts on the dipeptidyl-peptidase 4 (DDP-4) enzyme, responsible for indirect control of glycemia via the effect on incretins, regulators of insulin, and glucagon levels. Some divergent results were obtained; however, when compared with the positive control, sitagliptin, a conventional drug-inhibitor of DDP-4, it was demonstrated that *Cistus* extracts are weak DPP-4 inhibitors.

### 2.6. Cistus Biological Activity–Chemometric Approach

**Figure 3:**
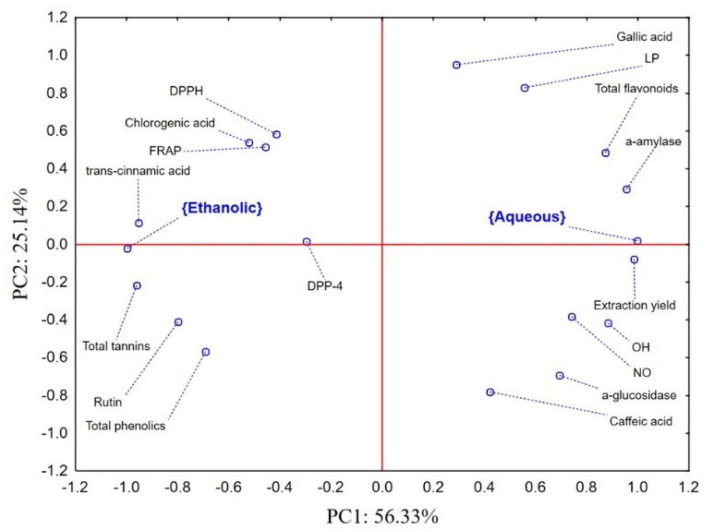
Principal component analysis (PCA) Biplot.

The application on principal component analysis (PCA) on a dataset containing the results of preliminary and detailed chemical characterization, as well as the results of biological potential of evaluated *Cistus* extracts revealed that the first two principal components (PCs) describe more than 80% of the samples’ variability. The position of the examined groups of samples (ethanolic and aqueous extracts) and evaluated variables in the space defined by the first two PCs shows a separate grouping of ethanolic and aqueous extracts (Figure 3).

Namely, in the positive part of the PC1 aqueous extracts characterized by a higher extraction yield, higher total flavonoid content, as well as higher caffeic and gallic acid contents were localized. Furthermore, aqueous extracts are more efficient scavengers of DPPH radicals. On the other hand, ethanolic extracts expressed better antioxidant potential in other evaluated assays. Furthermore, ethanolic extracts expressed more a prominent inhibitory potential of α-amylase and α-glucosidase. Taking into account the inversely proportional nature of variables describing IC_50_ values and the space defined by PC2, it can be concluded that the recorded anti-α-glucosidase potential mostly correlates to the quantified amounts of total phenolics and rutin, while the stronger inhibition of α-amylase is a result of higher levels of chlorogenic and *trans*-cinnamic acids.

## 3. Discussion

The results of the preliminary chemical characterization of aqueous and ethanolic extracts were compared with previously published results. A study investigating aqueous and methanolic extracts of *C. salviifolius* also detected a high phenolic content, additionally suggesting that use of methanol as a solvent results in a higher content of extracted phenolic compounds (408.43 mg gallic acid equivalents (GAE)/g dry extract (DE)) [11]. Another study, comparing methanol, butanol and water used as solvents, also concluded that methanol is the best solvent for the extraction of phenolic compounds [12]. However, it should be noted that methanol is considered to be a toxic solvent for human use, and the advantage should be given to ethanol when it comes to application in humans. One more study compared the leaves and flower buds, detecting a higher content of phenolics in the flower buds [13]. Other studies reported a low content of total phenolic compounds. This could potentially be explained by a shorter time of extraction [14], and in room temperature [15].

The present study determined that more than half of the total phenolics content is of tannin origin. This is consistent with one previously published result [11], and in contrast to another, where only the content of condensed tannins was determined [13]. Potentially, the high content of tannins could at least partially explain and justify the traditional application of this herb in the treatment of diarrhea, as tannin-rich herbs are often used in these cases [2].

The present study suggests that extraction with water results in a somewhat higher content of flavonoids. However, one study found there was no difference in the amount of extracted flavonoids when comparing the aqueous and methanolic extracts [14]. Previous studies reported contrasting data on the flavonoids content. A lower content of flavonoids has been previously reported in research where an aqueous extract was also prepared [15]. When methanol was used as a solvent for extraction, the reported content of flavonoids was up to 188.66 mg of rutin equivalents/g of DE [11]. A high content of flavonoids (up to 393 mg of quercetin equivalents (QE)/g DE, when aqueous extract was prepared from flowers of *C. salviifolius*), even higher than the content of phenolic compounds was reported in both flowers and leaves of *C. salviifolius* from Jordan, in all types of extracts (methanolic, butanolic and aqueous) [12].

Several studies evaluating the detailed chemical composition of various *C. salviifolius* extracts are also reported. The current study tested both types of extracts (aqueous and ethanolic) from both locations, revealing similarities between the locations. As seen in Table 2, the most abundant component was gallic acid. This is consistent with the previously determined high content of tannins, bearing in mind that gallic acid is a precursor in the biosynthesis of hydrolysable or pyrogallol-type tannins. Another study, investigating the chemical composition of the aqueous extract of this plant, also reported the presence of gallic acid, together with punicalin, punicalagin, punicalagin-gallate, and 7-xyloside ellagic acid. The same study quantified ellagitannins as punicalagin equivalents (approximately 15 mg/mL of extract) [16]. The presence of gallic acid was also reported in another study, in addition to the presence of shikimic and quinic acid, later reported to be the most abundant among the three compounds. Previously mentioned compounds (punicalin, punicalagin, punicalagin-gallate) were also detected [15]. Chlorogenic acid was detected in all four extracts, in lower amounts. Previous research rarely tested for this compound, although it was found in samples originating from Croatia [17]. HPLC-DAD analysis did not detect rosmarinic acid. Although this compound is most frequently, but not exclusively, found in Lamiaceae species, the present study opted for testing the presence of this valuable compound with high biological and pharmacological potential for the first time, to the best of our knowledge [18].

The second-most-abundant compound in the present study was rutin (also called rutoside, quercetin-3-O-rutinoside, and sophorin). Interestingly, this flavonol glycoside was not detected in aqueous extracts originating from Spain [16]. Another study, also conducted on Spanish samples, did not test the presence of rutin [15]. Quercetin, the aglycon of rutin, was not detected in the present samples, possibly due to the fact that no hydrolysis of the samples prior to chromatographic analysis was performed. Various glycosides of quercetin have been previously reported to be present, e.g., glucoside [15,16,17], pentoside, rhamnoside, and acetylhexoside [17]. Quercitrin was not detected in the present samples, which is consistent with previous results [16].

Several previous studies investigated the composition of EOs from various geographical locations. A sample of EO from the island of Cos also had germacrene D and (E)-β-farnesene as the two most abundant components in the present samples. The detected content of germacrene D was even higher, 51.55% [19]. Contrasting, a sample from Sicily had lower content of germacrene D (9.1%), although this was found to be the main compound in EO, followed by caryophyllene oxide [20]. Demetzos et al., 2002 [21] tested 15 samples from the Greek island of Crete, revealing that sesquiterpenes, especially oxygenated, were the main ingredients of EOs. Still, the most frequent compound in all samples was camphor, ranging up to 47.2% in one sample [21]. This compound was found in dried leaves of our present samples, and its content was lower in EOs obtained by hydrodistillation. Camphor was also the main compound detected as VOC in the samples originating from Spain (43.86%), where analysis was performed using solid-phase microextraction, a green method which eliminates the need for highly increased temperature necessary for EO isolation. Interestingly, the second-most-abundant compound was found to be eucalyptol (1,8-cineole) [22]. Solid-phase microextraction was also performed in another study, where the contents of VOCs were compared to those in EO, this being the most similar to the approach used in present research. While monoterpene hydrocarbons shared approximately 50% of the total analyzed VOCs content in both leaves and flowers, EO was characterized by the presence of diterpenes [12]. This group of compounds is considered as rarely present in EOs, although it has been previously reported in *C. salviifolius* samples [21,23], and even considered as a chemotaxonomic character [21]. Samples from Sardinia were characterized by a high content of norisoprenoids, while the main component of EO was β-damascenone [23]. Samples originating from Croatia showed that the main components found in EO were non-terpene compounds, with pentadecanoic (18.1%) and hexadecanoic acid (14.1%) being the main ones [24]. Furthermore, a study of *C. salviifolius* samples from the Elba island concluded that non-terpene compounds are more abundant in samples from non-contaminated areas (while phenylpropanoids are the main compounds in samples from former mining sites) [25].

Herbal extracts are often tested for their antioxidant potential. In vitro assays include several tests, with the DPPH test being the most common, because of its general robustness. Based on the present results, *C. salviifolius* was found to be highly active in the neutralization of DPPH radical formation. Samples from Tunisia showed similar activity as the present samples, where RSC_50_ obtained for the flower buds extract was 5.11 mg/L and for the leaves extract was 6.48 mg/L [13]. Sayah et al., 2017 [11] found similar values when both aqueous and methanolic extracts of aerial parts were tested (4.1 and 3.3 μg/mL, respectively). Samples originating from Croatia were found to be less active in this test [17]. Since DPPH is a synthetic radical and cannot be found in the human body [26], the present study also evaluated the potential of extracts to inhibit the formation of hydroxyl (OH) and NO radicals. To the best of our knowledge, previous studies have not tested the antioxidant potential in these tests. Thus, a comparison of *Cistus* extracts to positive controls was performed in corresponding tests for the first time. Based on the determined values, presented in Table 4, it can be concluded that *C. salviifolius* extracts are weak scavengers of OH radicals, while relatively effective neutralizers of the NO radicals formation. A test of lipid peroxidation (LP) process inhibition found divergent values of all four tested samples; however, the determined enzyme inhibitory potential (IC_50_) for butylated hydroxytoluene (BHT), suggests that *C. salviifolius* is a weak inhibitor of lipid peroxidation. However, different plant derived products, with less toxicity and side effects than synthetic antioxidants, especially BHT [27], nowadays play an important role in the prevention of various diseases and syndromes where reactive oxygen species or LP are involved, as well as natural preservatives in different pharmaceutical, food and cosmetic products. Additionally, ferric ion reduction (FRAP) tests were also previously performed on *C. salviifolius* samples. Activity recorded in the present extracts was found to be similar to the previously reported activity of aqueous and methanolic extracts [11]. Carev et al., 2020 [17] concluded that the aqueous extract was more potent than ascorbic acid, as determined in reported testing.

The treatment of (pre)diabetic conditions can include various remedies, where one of the stated mechanisms of activity is the inhibition of enzymes responsible for the digestion of complex carbohydrates (e.g., α-amylase and α-glucosidase). One previously reported study evaluated the antihyperglycemic effects of the *C. salviifolius* aqueous and methanolic extracts. Aqueous ones were revealed to be more potent inhibitors of enzymes α-amylase and α-glucosidase than the positive control, acarbose. Concerning the methanolic extract, it was more potent than acarbose in α-glucosidase inhibition, yet weaker in α-amylase inhibition [11]. Besides the inhibition of enzymes important for sugar metabolism and maintaining euglycemic levels, an in vitro study tested the aqueous extract, revealing its activity on the peroxisome proliferator-activated receptor γ and impact on glucose uptake by adipocytes. Investigation of the chemical compounds responsible for these activities demonstrated the importance of several flavonoid derivates and *trans*-cinnamic acid [28]. Interestingly, both ethanolic extracts presented in this study, which contained *trans*-cinnamic acid, had the best impact on α-amylase and α-glucosidase, possibly demonstrating the importance of this compound in maintaining the homeostasis of blood sugar levels via different mechanisms. One in vivo study tested the activity of *C. salviifolius* in diabetic mice. The aqueous extract successfully reduced the levels of blood sugar and total cholesterol in diabetic animals. Furthermore, it is confirmed that the extract reduced the harmful effects induced by streptozocin [10]. All of the previous studies, as well as the present work justify the traditional application of *Cistus*-based teas and ethanolic extracts the in treatment of (pre)diabetic conditions [2].

This study (together with previously published studies) reported a high content of tannins, which suggests an undesirable, astringent taste of preparations made in traditional forms (e.g., tea, drops, tincture, etc.). This can potentially affect the intake of the herb in these forms. As high potency of extracts (aqueous and ethanolic, and essential oil) is presented here, the future steps in the application of *Cistus salviifolius* would include formulating the adequate dosage form which would annihilate the repugnant palatability. Potentially, the pharmaceutical dosage form (e.g., capsules containing aqueous/ethanolic extracts) would be of the greatest benefit for patients with metabolic disorders of carbohydrates, at the same time ensuring the compliance of these patients.

The presented study has several limitations. The herbal samples were collected from only two locations, and the impact of ecological abiotic factors (such as temperature, soil composition, insolation, etc.) was not investigated. Additionally, the safety of used herbal material was not investigated, primarily in the form of testing the samples for heavy metals and organic contamination, as it was previously described that this herbal species can accumulate these toxic matters. However, this study also has several strengths. The study has given more detailed data on the activity in antioxidant tests, bearing in mind that it utilized five different, yet complementary systems, while other studies usually refer only to DPPH radicals scavenge capacity testing. Additionally, the antihyperglycemic activity was tested in inhibition tests for three enzymes, again being more complete than previously reported studies.

## 4. Materials and Methods

### 4.1. Herbal Material

**Figure 4:**
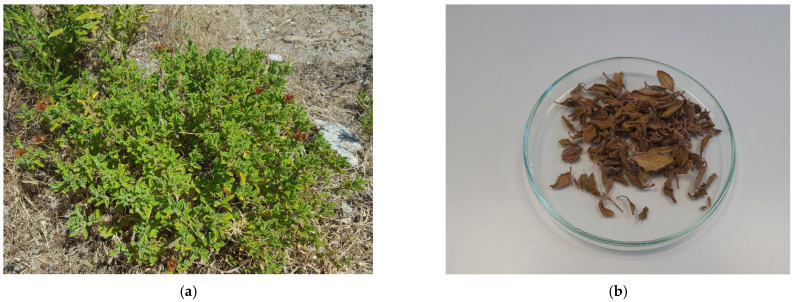
(**a**) *Cistus salviifolius* at location Petaloudes; (**b**) the appearance of the herbal drug.

Plant samples were collected at two locations, Petaloudes (Figure 4a) and Epta Piges, Rhodes, Greece, in August of 2019. Voucher specimens (2-1430 and 2-1431), confirming the identity, were deposited at the Herbarium of the Department of Biology and Ecology (BUNS Herbarium), Faculty of Sciences, University of Novi Sad. Herbal drugs used in the experiments consisted of dried leaves (Figure 4b), which were pulverized using pulse grinding with an electric mill before biochemical characterization.

### 4.2. Preparation of Samples for Further Analysis

Two types of extracts were prepared: aqueous and ethanolic. In both cases, the proportion of drug:solvent was 1:10 (3 g of herbal drug was mixed with 30 mL of water or 30 mL of ethanol). The aqueous extract was prepared by decoct for 30 min, while ethanolic extraction was performed as a maceration at room temperature for 24 h where 70% (*w*/*w*) ethanol was used as a solvent. In both cases, after the removal of solvents by evaporation, the content of DE was determined.

For the purposes of chemical characterization by the means of HPLC-DAD, DEs of both types of extracts were dissolved in methanol.

As herbal materials were additionally analyzed for VOCs content, samples were submitted separately to two different procedures. The EOs were isolated from dried leaves by hydrodistillation according to European Pharmacopoeia 10 [29] in Clevenger apparatus for 2 h, using n-hexane as the collecting solvent, which was further removed in vacuo and the yield of essential oil was determined gravimetrically. Prior to EO chemical analysis by GC-MS technique it was diluted by 1 mL of n-hexane Furthermore, dried leaves of both samples of *C. salviifolius* were crushed in headspace vials and hermetically sealed before the analysis for profiling and quantification of VOCs by the means of headspace sampling (HSS).

Samples were marked according to the location of herbal samples (P or E, for Petaloudes or Epta Piges, respectively) and type of extract (Aq or Et, for aqueous or ethanolic, respectively or HSS or EO, for headspace sampling or essential oil, respectively).

### 4.3. Chemical Characterization

#### 4.3.1. Preliminary Chemical Characterization

Preliminary chemical analysis included the quantification of total phenolics and flavonoids as previously described [30]. The content of total tannins was determined indirectly after precipitation by an adsorbent Hide Powder (Sigma, Burlington, MA, USA), according to the procedure described in European Pharmacopoeia 10 [29]. The content of total flavonoids was expressed as mg of QE per gram of DE (mg QE/g DE), while the content of total phenolics and total tannins was expressed as mg of GAE per gram of dry extract (mg GAE/g DE).

#### 4.3.2. HPLC-DAD Chemical Profiling

Detailed chemical characterization was performed by HPLC-DAD, according to a previously described method [31]. The analysis was performed on Agilent HP 1100 with the DAD system equipped with an autosampler (Agilent Technologies, Waldbronn, Germany). The compounds of interest were separated on a Nucleosil C18 column (5 μm × 4.6 mm × 250 mm,) held at 30 °C. The mobile phase A consisted of 1% (*V*/*V*) aqueous solution of formic acid, while mobile phase B was methanol; gradient elution was performed according to the following program: 0–10 min, 10–25% B; 10–20 min, 25–45% B; 20–30 min, 45% B; 30–35 min, 45–70% B; 35–40 min, 70–100% B; 40–43 min, 100% B. Ten selected compounds, present in the plant species and significant for their biological and pharmacological potential were screened—seven phenolic acids, namely gallic, *trans*-cinnamic, *p*-coumaric, caffeic, ferulic, chlorogenic and rosmarinic acid, and three flavonoids, aglycon quercetin and two glycoside forms, quercitrin and rutin. All the used standards were of HPLC quality (manufacturer Sigma Aldrich). The chromatograms were monitored at 280 nm for gallic, caffeic and *trans*-cinnamic acids, 330 nm for *p*-coumaric, chlorogenic, rosmarinic and ferulic acids and quercetin, and 350 nm for rutin and quercitrin. The content of the compounds was expressed as mg/g of DE.

### 4.4. Chemical Analysis of Volatile Organic Compounds

The chemical characterization of VOCs was performed using gas chromatography (GC; Agilent 6890B GC-FID instrument, Waldbronn, Germany) coupled to a mass spectrometry detector (MSD; Agilent 5977 MSD, Waldbronn, Germany) on a HP-5MS capillary column (30 m × 0.25 mm, 0.25 μm). In both types of prepared samples (HSS and EO), the samples were injected in splitless mode (purge flow to split vent: 15 mL/min at 0.75 min) and at inlet temperature of 220 °C. The initial oven temperature was set at 60 °C and increased at a rate of 3 °C/min until reaching 246 °C. The carrier gas was helium, with the flow rate of 1 mL/min. The temperature of MSD transfer line was set to 230 °C. Data were collected in scan mode (*m*/*z* = 50–550). Mass spectral database NIST (v14), as well as literature data [32] were used for VOC identification.

### 4.5. Antioxidant Potential

Concerning the wide variety of reactive chemical entities occurring in an organism and their possible modes of action, it is highly recommended to perform several different assays in order to obtain more comprehensive data about the antioxidant potential of specific agents. Therefore, the potential of analyzed herbal extracts to neutralize free radicals (DPPH, OH, and NO radicals), inhibit the LP process, as well as to perform FRAP was estimated spectrophotometrically. Briefly, a range of herbal extracts concentrations was added to a solution of corresponding free radicals. The reduction of DPPH radical was measured spectrophotometrically at 515 nm. In the OH test, free radicals were generated in a Fenton reaction and the degradation of 2-deoxy-D-ribose to malonyl-dialdehyde (MDA) was monitored spectrophotometrically at 532 nm. Similarly, the inhibition of the lipid peroxidation process was estimated based on the potential of examined extracts to protect the liposomes, as substrate, from free radicals generated in a Fenton reaction and the content of formed MDA was determined at 532 nm. On the other hand, the donor of NO radicals was sodium nitroprusside, while the appearance of the purple complex with Greiss reagent was detected spectrophotometrically at 546 nm. The tests were performed according to a previously described methodology [30,33]. The ability to reduce ferric to ferrous salt was estimated on three concentration levels, as previously described [34,35]. As positive controls, established antioxidants were used, namely, BHT in OH and LP tests, ascorbic acid (AA) in the OH test, and propyl gallate (PG) in DPPH, OH, and NO tests. All assays were performed in triplicates. The results of the antioxidative potential evaluation were expressed as the concentrations of the extract required to neutralize 50% of the free radicals (RSC_50_, in tests of NO, OH, and DPPH radical neutralization) or as concentrations required for the inhibition of 50% of the oxidative process (IC_50_, in test of lipid peroxidation inhibition). The FRAP assay results were expressed as milligrams of the ascorbic acid equivalents per gram of DE (mg AAE/g DE).

### 4.6. Antihyperglicemic Potential

The antihyperglycemic potential of the examined extracts was evaluated through the inhibition of three enzymes included in the initial metabolism of carbohydrate digestion—α-amylase, α-glucosidase, and DPP-4. Briefly, the increasing concentrations of *Cistus* extracts were added to the reaction mixture containing porcine α-amylase (final activity 0.6 U/mL), modified starch with an indicator (Starch Azure) and a phosphate buffer (pH = 7.2). The α-glucosidase test’s assay mixture contained an enzyme originating from *Saccharomyces cerevisiae* (final activity 7.6 U/L), glutathione (reduced form), p-nitrophenyl-α-D-glucoside (PNP-Gluc) (as a substrate) and a phosphate buffer (pH = 6.8). Acarbose was used as a positive control in both of the previously mentioned assays [33]. The potential of the examined extracts to inhibit the DPP-4 activity was evaluated spectrophotometrically, according to a previously described kinetic method [36]. The reaction mixtures contained the DPP-4 enzyme solution (final concentration 0.025 U/mL) in a Tris buffer and increasing concentrations of herbal extracts. After incubation at 37 °C for 20 min, the reaction was started by the addition of a substrate (Gly-Pro-p-nitroanilide) and the change of absorption at 405 nm was monitored spectrophotometrically for 3 min. Sitagliptin was used as a positive control. All tests were performed in triplicates. The herbal extracts’ inhibitory potential was expressed as a concentration of extract required to inhibit 50% of enzyme activity (IC_50_).

### 4.7. Data Analysis

The results obtained in the study were processed by MS Office Excel (v2019) and Statsoft Statistica (v12.5) software packages. Data were processed by means of univariate (descriptive) and multivariate statistical analysis. The univariate differences between evaluated samples were analyzed by the application of one-way ANOVA followed by Bonferroni post-hoc test, while the differences were considered significant if *p* < 0.05. The PCA was applied on dataset describing chemical profile, antioxidant, and antihyperglycemic potential of the examined samples.

## 5. Conclusions

*Cistus salviifolius* is a relatively poorly investigated herbal species. The present results suggested a high abundance in the phenolic type of secondary metabolites, especially tannins, as well as the presence of an essential oil rich in sesquiterpene hydrocarbons. This implicates the high utilization potential of this plant as a source of biologically active compounds. The obtained extracts were screened in for antioxidant and antihyperglycemic activities. The recorded antioxidant potential was classified as highly prominent, especially in tests of DPPH and NO radical neutralization. Extracts also demonstrated high anti-α-amylase and anti-α-glucosidase potency, while the recorded inhibition potential is presumed to be directly correlated with the content of chlorogenic and *trans*-cinnamic acids, and total phenolics and rutin, respectively. However, further studies on different biological effects in vitro and on cell lines, as well as on experimental animals and in human subjects, are required in order to evaluate the possibility of its inclusion as a prevention or complementary therapy in some diseases and conditions.

## Figures and Tables

**Table 1:** Results of the preliminary chemical analysis.

	P-Et	E-Et	P-Aq	E-Aq
yield of extraction (%)	13.45 ± 0.95 ^a,b^	14.84 ± 0.76 ^c^	37.04 ± 1.22 ^a,c^	33.32 ± 1.11 ^b,c^
content of total phenolics (mg GAE/g DE)	352.88 ± 3.56 ^d^	379.58 ± 0.88 ^d,e^	352.37 ± 3.27 ^e^	279.32 ± 0.33 ^d,e^
content of total tannins (mg GAE/g DE)	301.30 ± 4.44 ^f^	329.49 ± 2.78 ^f^	211.18 ± 8.11 ^f^	159.22 ± 6.03 ^f^
content of total flavonoids (mg QE/g DE)	26.33 ± 0.87 ^g,h^	29.41 ± 1.43 ^i^	36.20 ± 2.98 ^g,j^	45.37 ± 4.02 ^h,i,j^

P—Petaloudes location, E—Epta Piges location, Et—ethanolic extract, Aq—aqueous extract, GAE—gallic acid equivalents, DE—dry extract, QE—quercetin equivalents. The same Latin letters denote statistically significant differences.

**Table 2:** Results of high-performance liquid chromatography coupled to diode array detector (HPLC-DAD) analysis, determining the content of 10 selected compounds.

	P-Et	E-Et	P-Aq	E-Aq
gallic acid (mg/g DE)	14.96 ± 2.244	21.18 ± 3.177	15.09 ± 2.263	27.67 ± 4.15
*trans*-cinnamic acid (mg/g DE)	0.14 ± 0.015	0.24 ± 0.026	n.d.	n.d.
*p*-coumaric acid (mg/g DE)	n.d.	n.d.	n.d.	n.d.
caffeic acid (mg/g DE)	0.43 ± 0.021	0.46 ± 0.023	0.63 ± 0.032	0.41 ± 0.021
ferulic acid (mg/g DE)	n.d.	n.d.	n.d.	n.d.
chlorogenic acid (mg/g DE)	0.95 ± 0.047	2.20 ± 0.11	0.89 ± 0.045	1.28 ± 0.064
rosmarinic acid (mg/g DE)	n.d.	n.d.	n.d.	n.d.
quercetin (mg/g DE)	n.d.	n.d.	n.d.	n.d.
quercitrin (mg/g DE)	n.d.	n.d.	n.d.	n.d.
rutin (mg/g DE)	8.97 ± 0.718	5.59 ± 0.447	4.09 ± 0.237	2.58 ± 0.206

P—Petaloudes location, E—Epta Piges location, Et—ethanolic extract, Aq—aqueous extract, DE—dry extract, n.d.—not detected.

**Table 3:** The results of volatile organic compounds content analysis in dried leaves and essential oil of *Cistus salviifolius* (the results are expressed in percentages as mean values ± standard deviation of three replicates).

Peak No.	Compound	RI	RI^l^	P-HSS	E-HSS	P-EO	E-EO
**Monoterpene hydrocarbons**				**5.54**	**6.45**	**0.42**	**0.90**
1	α-Pinene	939	933	0.86 ± 0.04	0.65 ± 0.03	0.47 ± 0.02	0.62 ± 0.03
2	β-Pinene	979	973	2.91 ± 0.14	3.3 ± 0.16	0.42 ± 0.02	0.28 ± 0.01
4	*o*-Cymene	1025	1025	0.96 ± 0.05	1.39 ± 0.07	n.i.	n.i.
5	Limonene	1029	1023	1.67 ± 0.08	1.76 ± 0.09	n.i.	n.i
**Oxygenated monoterpenes**				**5.27**	**7.82**	**3.05**	**1.14**
7	Camphor	1146	1129	5.02 ± 0.25	7.63 ± 0.38	1.11 ± 0.05	0.88 ± 0.04
8	Carvenone	1258	1248	0.25 ± 0.01	0.19 ± 0.01	1.94 ± 0.10	0.26 ± 0.01
**Aromatic oxygenated monoterpenes**				**2.62**	**2.63**	**0.96**	**3.69**
9	Thymol	1291	1275	2.62 ± 0.13	2.63 ± 0.13	0.96 ± 0.05	3.69 ± 0.18
**Sesquiterpene hydrocarbons**				**67.82**	**65.30**	**79.00**	**77.02**
10	α-Cubebene	1351	1530	n.i.	n.i.	0.55 ± 0.03	0.34 ± 0.02
11	α-Copaene	1377	1376	n.i.	n.i.	0.63 ± 0.03	0.8 ± 0.04
12	β-Elemene	1395	1374	0.91 ± 0.04	0.85 ± 0.04	0.95 ± 0.05	0.63 ± 0.03
13	Longifolene	1408	1401	1.74 ± 0.09	1.81 ± 0.09	2.18 ± 0.11	0.35 ± 0.02
14	E-caryophyllene	1419	1419	2.19 ± 0.11	2.48 ± 0.12	2.55 ± 0.13	3.52 ± 0.18
15	β-Copaene	1432	1434	0.92 ± 0.05	0.46 ± 0.02	4.53 ± 0.23	4.66 ± 0.23
16	(E)-β-Farnesene	1456	1448	19.67 ± 0.98	18.23 ± 0.91	16.39 ± 0.82	17.34 ± 0.87
17	Alloaromadendrene	1461	1460	n.i.	0.11 ± 0.01	0.85 ± 0.04	0.36 ± 0.02
18	β-Selinene	1486	1481	0.89 ± 0.04	0.62 ± 0.03	0.89 ± 0.04	0.79 ± 0.04
19	Germacrene D	1488	1485	40.92 ± 2.04	39.82 ± 1.99	41.092.05	39.78 ± 1.99
20	epi-Bicyclosesquiphellandrene	1496	1490	n.i.	0.12 ± 0.01	1.25 ± 0.06	1.77 ± 0.09
21	α-Muurolene	1499	1495	n.i.	n.i.	0.83 ± 0.04	0.81 ± 0.04
22	Bicyclogermacrene	1502	1497	0.23 ± 0.01	0.36 ± 0.02	4.47 ± 0.22	3.45 ± 0.17
23	β-Bisabolene	1511	1505	0.35 ± 0.02	0.44 ± 0.02	0.65 ± 0.03	0.83 ± 0.04
24	(-)-β-Cadinene	1518	1522	n.i.	n.i.	1.19 ± 0.04	1.59 ± 0.08
**Aromatic sesquiterpene hydrocarbons**				**1.46**	**1.47**	**3.39**	**3.06**
25	cis-Calamenene	1531	1531	1.24 ± 0.06	1.33 ± 0.07	2.11 ± 0.10	1.52 ± 0.08
26	α-Calacorene	1542	1540	0.22 ± 0.01	0.14 ± 0.01	1.28 ± 0.06	1.54 ± 0.08
**Oxygenated sesquiterpenes**				**10.71**	**11.53**	**11.16**	**9.95**
27	(-)-Spathulenol	1577	1568	0.32 ± 0.02	0.23 ± 0.01	1.54 ± 0.08	1.44 ± 0.11
28	Caryophyllene oxide	1581	1574	8.29 ± 0.41	9.77 ± 0.49	6.28 ± 0.31	5.66 ± 0.28
30	α-Bisabolol oxide	1744	1722	1.98 ± 0.10	1.53 ± 0.08	2.66 ± 0.13	2.19 ± 0.11
31	b-Bisabolenol	1790	1774	0.12 ± 0.01	n.i.	0.68 ± 0.03	0.66 ± 0.03
**Aliphatic compounds**				**2.00**	**2.80**	**n.i.**	**n.i.**
3	n-Decane	1000	1015	0.87 ± 0.04	0.97 ± 0.05	n.i.	n.i.
6	Undecane	1100	1115	0.98 ± 0.05	1.52 ± 0.08	n.i.	n.i.
29	Heneicosane	1600	2100	1.02 ± 0.05	1.28 ± 0.06	n.i.	n.i.
**TOTAL OF INDENTIFIED COMPOUNDS**				**95.42**	**98.00**	**97.98**	**95.76**

RI—retention index, RI^l^—retention index reported in literature, P—Petaloudes location, E—Epta Piges loaction, HSS—headspace sampling, EO—essential oil, n.i.—not identified.

**Table 4:** The results of antioxidant potential testing.

	P-Et	E-Et	P-Aq	E-Aq	BHT	AA	PG
DPPH (RSC_50_, in μg/mL)	1.98 ± 0.08 ^a^	1.78 ± 0.1 ^b^	1.54 ± 0.04 ^a,b,c^	1.92 ± 0.06 ^c,d^	/	/	0.59 ± 0.04 ^a,b,c,d^
OH (RSC_50_, in μg/mL)	n.d.	n.d.	145.17 ± 5.92 ^e^	66.27 ± 3.33 ^e,f^	0.04 ± 0.01 ^e,f^	2.09 ± 0.11 ^e,f^	9.1 ± 0.52 ^e,f^
NO (RSC_50_, in μg/mL)	11.14 ± 0.98	10.07 ± 0.85	11.48 ± 0.44	11.24 ± 1.01	/	/	9.23 ± 0.39
LP (IC_50_, in μg/mL)	26.56 ± 1.11 ^g^	38.66 ± 2.83 ^g,h^	33.45 ± 2.84 ^i^	68.71 ± 5.55 ^g,h,i,j^	7.45 ± 0.55 ^g,h,i,j^	/	/
FRAP (mg AAE/g DE)	628.10 ± 3.76 ^k^	679.74 ± 4.67 ^k,l^	631.15 ± 2.33 ^l^	643.75 ± 2.94 ^k,l^	/	/	/

P—Petaloudes location, E—Epta Piges location, Et—ethanolic extract, Aq—aqueous extract, BHT—butylated hydroxytoluene, AA—ascorbic acid, PG—propyl gallate, DPPH—2,2-diphenyl-1-picrylhydrazil, RSC_50_—concentration which can scavenge 50% of radicals, OH—hydroxyl radical, n.d.—not determined in tested concentrations, NO—nitroso radical, LP—lipid peroxidation, IC_50_—concentration which inhibits 50% of enzyme’s activity, FRAP—ferric reducing antioxidant power, AAE—ascorbic acid equivalents, DE—dry extract. The same Latin letters denote statistically significant differences.

**Table 5:** The results of antihyperglycemic potential testing.

	P-Et	E-Et	P-Aq	E-Aq	Acarbose	Sitagliptin
α-amylase (IC_50_, in μg/mL)	3.46 ± 0.22 ^a^	5.44 ± 0.42 ^b^	17.59 ± 0.99 ^a,b,c^	23.79 ± 1.09 ^a,b,c,d^	4.23 ± 0.33 ^c,d^	/
α-glucosidase (IC_50_, in μg/mL)	0.098 ± 0.01 ^e^	0.066 ± 0.01 ^f^	0.33 ± 0.02 ^g^	0.12 ± 0.04 ^h^	44.67 ± 1.22 ^e,f,g,h^	/
DPP-4 (IC_50_, in μg/mL)	1124.58 ± 5.67 ^i^	313.30 ± 4.02 ^i,j^	316.98 ± 8.41 ^i,k^	651.56 ± 4.38 ^i,l^	/	0.0211 ± 0.0016 ^i,j,k,l^

P—Petaloudes location, E—Epta Piges location, Et—ethanolic extract, Aq—aqueous extract, IC_50_—concentration which inhibits 50% of enzyme’s activity, DPP-4—dipeptidyl-peptidase 4. The same Latin letters denote statistically significant differences.

## Data Availability

Not applicable.

## Sample Availability

Samples of herbal drugs are available from the authors.
